# Negative association between harsh parenting and life satisfaction: negative coping style as mediator and peer support as moderator

**DOI:** 10.1186/s40359-023-01046-0

**Published:** 2023-01-20

**Authors:** Chensen Ma, Jingjing Song

**Affiliations:** 1 Tongji Hospital, Tongji Medical College, Huazhong University of Science and Technology, Wuhan, People’s Republic of China; 2 School of Education, Institute of Psychology, China University of Geosciences, 388 Lumo Road, Wuhan, 430074 People’s Republic of China

**Keywords:** Harsh parenting, Negative coping style, Life satisfaction, Peer support

## Abstract

**Background:**

Previous research has demonstrated that harsh parenting negatively affects children’s psychological development. This study examined the association between harsh parenting during childhood and life satisfaction of Chinese college students. We further looked at whether this association is explained in part by negative coping styles, and whether peer support lessens the potential effect of harsh parenting on negative coping styles and life satisfaction.

**Method:**

The sample included 609 Chinese students (aged 17–21 years, *M* = 18.39, *SD* = 0.82). The participants responded to questionnaires measuring past experiences with harsh parenting, life satisfaction, negative coping styles, and peer support.

**Results:**

Regression analysis showed that harsh parenting negatively contributed to students’ life satisfaction via the mediator of negative coping styles, and peer support moderated this negative relationship. Specifically, the negative impact of harsh parenting on life satisfaction was only significant when there was low peer support. The effect of harsh parenting on negative coping styles was higher in individuals with high peer support than in those with low peer support.

**Conclusion:**

This study highlights the roles of intrinsic (negative coping style) and extrinsic (peer support) factors in understanding the negative effects of harsh parenting on adolescents’ life satisfaction. These results provide insight into how to enhance adolescents’ life satisfaction by reducing harsh parenting and negative coping styles and by promoting peer support.

## Introduction

Life satisfaction refers to an individual’s self-evaluation of how satisfied they are with their own life [1]. It is significantly positively correlated with mental health status and negatively correlated with depression [2]. It is necessary to pay attention to factors that influencing life satisfaction [3]. Based on the bio-ecological model of human development, family situation and parenting style are the most important factors influencing children’s emotional and physical development [4]. Harsh parenting, the most negative parenting style, affects children’s psychological life satisfaction [5]. Harsh parenting refers to physical and verbal aggression against a child, including behaviors such as yelling, slapping, spanking, shoving, or beating the child with an object [6, 7].

Harsh parenting is common in China. Chen investigated 185 Chinese parents and found that 52% had physically abused their children [8]. Another investigation focused on 1164 Chinese parents and found that 78.1% had psychologically abused their children in the last three months [9]. Our study analyzed the negative association between harsh parenting during childhood and life satisfaction of Chinese college students, and further explored the mechanisms involved.

### Harsh parenting and life satisfaction

We assumed that harsh parenting during childhood would be negatively associated with life satisfaction of Chinese college students. Prior research has demonstrated that harsh parenting is an important risk factor in the psychosocial development of Chinese college students. Harsh parenting has been shown to be associated with Chinese adolescents’ insecure attachment and social anxiety [10], emotional dysregulation [11], poor peer relationships [12], less effortful control and higher aggression [7], more problematic Internet use [13], and poor academic performance [14]. To our knowledge, no study has directly analyzed the negative relationship between harsh parenting and life satisfaction of Chinese college students. The most similar study on this topic has been a study showing that authoritative parenting dimensions, including parental support, warmth, and autonomy, are positively associated with adolescents’ life satisfaction [5].

There may be cultural differences in parenting styles. The typical ways in which parents and children relate to or interact with each other are influenced by cultural factors [15]. Western parents pay considerable attention to the development of their children’s independence and self-realization [15, 16]; whereas, Asian parents emphasize the cultivation of family identity, a sense of belonging, and parental respect, along with the importance of hard work and self-discipline [15, 16]. Cultural differences may also exist in how these different parenting styles affect children’s development. The authoritative style has beneficial effects for European-American children. However, this has not always been the case with Asian-American children [15]. One study found that authoritarian parenting was positively related to the grades of Chinese children in Hong Kong [17]. Thus, we assumed harsh parenting might have a different effect on Chinese families compared with on American families. The universality of harsh parenting in the Chinese culture and its attribution style (the idea that parents are good for children) might lead to the negative effects of harsh parenting on Chinese children being less than those found in Western countries.

### The mediation role of negative coping styles

Negative coping styles might mediate the relationship between harsh parenting and life satisfaction. Harsh parenting is positively associated with children’s negative coping styles. Parents play an important role in children’s psychological and social development. Parents who use harsh parenting techniques have a negative coping style and poor emotional management ability. Therefore, children who have been harshly parented fail to learn adaptive coping strategies and emotion management methods. Additionally, they apply negative coping styles in response to stress, have difficulty in solving life problems, and have a greater possibility of immersed in negative emotion.

Previous research has confirmed the effect of parenting on coping styles [18, 19]. Authoritative parenting is associated with greater positive coping styles [19], whereas authoritarian parenting and parental psychological control is associated with higher levels of avoidant coping [20].

Meanwhile, negative coping styles may undermine life satisfaction. Researchers have observed that negative coping styles are negatively associated with life satisfaction [21] and subjective well-being [22]. Negative coping styles have also been correlated with mental health outcomes, such as negative automatic thoughts, stress, anxiety, and depression [23]. Thus, we assumed that negative coping styles plays a mediating role in the relationship between harsh parenting and life satisfaction.

### The moderating effect of peer support on the relationship between harsh parenting, coping styles, and life satisfaction

This study focuses on first-year Chinese college students. Late adolescence is a period in which children pursue independence and try to break away from their parents’ control. Adolescents spend a significant amount of time with their peers, and peers become increasingly important for their healthy growth [24]. Peer support refers to perceived emotional care, companionship, and help from friends [25]. Prior studies have confirmed that peer support, as an important environmental factor, can affect adolescents’ self-esteem, emotion regulation, resilience, mental capital, happiness, negative emotions (depression, anxiety, loneliness), and behavioral problems (aggression, early school dropout, problematic Internet use) [26, 27]. The bio-ecological model of human development indicates that individual development is simultaneously influenced by multiple risk factors such as family, peers, school, and community [4]. Therefore, in this study, we tested the simultaneous effects of early harsh parenting and current peer support on life satisfaction of Chinese college students.

Peer support might play a moderating role in the relationship between harsh parenting, negative coping styles, and life satisfaction. The stress-buffering effect model assumes that social support (including peer support) reduces the negative effects of stressful events (e.g., harsh parenting), decreases individuals’ perceptions and evaluations of stressful events as threatening, and reduces emotional and physiological reactions to stressful events. Further, social support promotes physical and mental health [28]. Previous research has also shown that interactions between the peer and family environments may predict adolescents’ behavior. One study noted that the promotion effect of poor peer communication on problematic behavior was higher in boys with low family management than in boys with high family management [29]. In another study, the relationship between parent-child attachment and psychological capital and with pro-social behavior was stronger for adolescents with low deviant peer affiliation than for those with high deviant peer affiliation [30]. Therefore, it is plausible that peer support moderates the relationships between harsh parenting, coping styles, and life satisfaction. We assumed that the effect of harsh parenting on negative coping styles and life satisfaction might be higher for individuals with low peer support than for those with high peer support.

### The current study

**Fig. 1:**
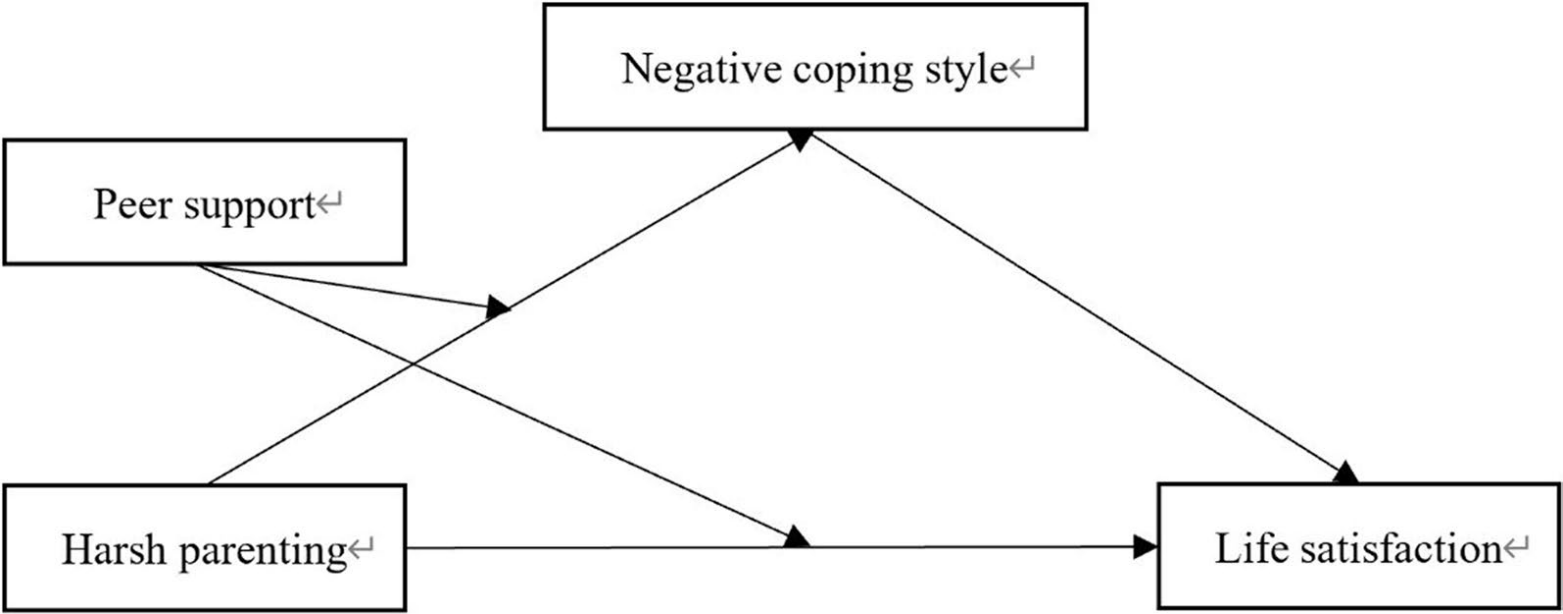
The effect of harsh parenting on late adolescents’ life satisfaction: A moderated mediation model

The purpose of this study was to test the direct and indirect effects of harsh parenting on life satisfaction of Chinese college students. We explored the mediating role of negative coping styles in the relationship between harsh parenting and life satisfaction and analyzed the moderating role of peer support in the mediation model. We hypothesized that negative coping styles would mediate the association between harsh parenting and life satisfaction of Chinese college students (H1). Specifically, harsh parenting would be positively associated with negative coping styles (H1a), and negative coping styles would be negatively associated with life satisfaction of Chinese college students (H1b). We also hypothesized that peer support would moderate the relationship between harsh parenting and life satisfaction (H2), as well as the relationship between harsh parenting and negative coping styles (H3). Figure 1 illustrates the moderated mediation model.

## Method

### Participants

We invited first-year college students from eight randomly selected mental health courses at a university in Central China. In total, 620 college students volunteered to participate. We eliminated data from 11 participants because their responses were same across most questions, suggesting that their responses were invalid. Among the remaining 609 participants, 344 were males (56.5%) and 265 were females (43.5%), aged 17–21 years (M = 18.39, SD = 0.82). There were 227 rural (37.3%) and 382 urban (62.7 %) areas represented by the locations of the students’ homes. In response to one question about their subjective family economic situations, 42 participants perceived their families as very poor (6.9%), 90 as a little poor (14.8%), 437 as average (71.8%), 37 as a little rich (6.1%), and 3 as very rich (0.5%).

### Procedure

Approval to conduct this study was obtained from the university’s ethics committee. Students completed the questionnaires before they take in the mental health courses. To standardize the data collection process, two trained research assistants introduced this investigation in accordance with the procedure manual. Participants were informed that their answers would be anonymous and were reassured that they could withdraw from the study at any time without penalty. After receiving a briefing of the study, the participants provided informed consent. Students who volunteered to participate scanned the QR code to obtain an online questionnaire. They took approximately ten minutes to complete all the questionnaires. The participants received partial credit for the course requirements.

### Measures

#### Harsh parenting

Harsh parenting was measured using the Harsh Parenting Scale [6]. In this study, we used the Chinese-language version of this measure, which is known to have high validity [7]. This scale included four items. An example item is: “In my childhood, when I did something wrong or made my parents angry, my parents lost their temper or even yelled at me.” The participants answered these four items on a five-point Likert scale (1 = never, 5 = always). Higher scores indicated higher perceived harsh parenting. The scale’s internal consistency was good for the current sample (Cronbach’s alpha = 0.89).

#### Negative coping styles

Negative coping styles were measured using the Coping Style Scale for Middle School Students [31]. The Scale had 30 items with two dimensions: positive and negative. We used only the negative coping styles subscale (17 items). Participants answered these items on a five-point Likert scale (1 = does not describe me at all; 5 = describes me very well). Higher scores indicated a higher level of negative coping styles. The scale’s internal consistency was good for the current sample (Cronbach’s alpha = 0.89).

#### Peer support

Peer support was measured using the Multidimensional Perceived Social Support Scale [32]. This 12-item self-report scale includes three dimensions: family, peer, and other support. In this study, we used four items to assess peer support. An example item is: “My friends are trying to help me.” Zhao and Li translated these items into Chinese [33], and the Chinese version of the peer support subscale showed good reliability and validity [34]. The participants answered these four items on a five-point Likert scale (1 = never, 5 = always). Higher scores indicated higher perceived peer support. The scale’s internal consistency was good for the current sample (Cronbach’s alpha = 0.94).

#### Life satisfaction

Life satisfaction was assessed using the Chinese version of the Life Satisfaction Scale [35]. Participants rated five items (e.g., “My life is going well”) on a five-point Likert scale (1 = does not describe me at all; 5 = describes me very well). Higher scores indicated higher levels of life satisfaction. The scale’s internal consistency was good for the current sample (Cronbach’s alpha = 0.90).

## Results

### Descriptive statistics and correlations among the variables

The correlations between harsh parenting, negative coping styles, peer support, and life satisfaction were calculated using Pearson’s product-moment correlation coefficient (see Table 1). The results indicated that harsh parenting was positively associated with negative coping styles and negatively associated with peer support and life satisfaction. Life satisfaction was negatively correlated with harsh parenting and negative coping styles, and positively associated with peer support.

**Table 1:** Descriptive statistics and correlation results among all variables

	1	2	3	4	5	6
Gender	–					
Age	0.09^*^	–				
Parenting	0.11^**^	0.07	–			
Coping style	0.01	0.02	0.26^***^	–		
Peer support	−0.13^***^	−0.01	−0.22^***^	0.02	–	
Life satisfaction	−0.08^*^	−0.08^*^	−0.22^***^	−0.18^***^	−0.31^***^	–
*M*	0.56	18.39	7.58	50.32	15.51	16.63
*SD*	0.50	0.82	3.49	10.39	3.35	4.40

The mean for gender is the percentage of male students. ^*^p < 0.05, ^**^p < 0.01, ^***^p < 0.001

The single-factor Harman test was used to assess common method variance. The results of the exploratory factor analysis showed that the first factor explained 24.28% of the variance (lower than the threshold of 40%), indicating that common method variance was not a serious threat in this study.

We used ANOVA to analyze gender differences in perceived harsh parenting, negative coping styles, peer support, and life satisfaction. We found significant gender differences in harsh parenting, life satisfaction, and peer support, F (1607) = 7.18, *p* < 0.01, partial η^2^ = 0.012; F (1607) = 4.10, *p* < 0.05, partial η^2^ = 0.007; F (1607) = 10.75, *p* < 0.001, partial η^2^ = 0.017. There were no significant gender differences in negative coping styles. Male students experienced more harsh parenting (M = 7.91), less life satisfaction (M = 16.31), and less peer support (M = 15.12) than female students (M = 7.15, M = 17.04, M = 16.01).

### The direct and indirect effect of harsh parenting on life satisfaction

We used the PROCESS macro in SPSS [36] to test the direct effect of harsh parenting on life satisfaction, the indirect effect via negative coping style, and the moderating effect of peer support (see Fig. 1). The moderated mediation model analysis was conducted using Model 8, as the moderator influenced the independent variable’s effect on the dependent variable and mediator. All predictors were standardized to minimize multicollinearity. Moreover, gender, family economic situation, and age were controlled for in this model. Bootstrapping with 5000 iterations was used to generate an approximation of the sampling distribution to obtain accurate confidence intervals.

The model which tested harsh parenting, peer support, and their interactions as predictors of negative coping styles was significant, R^2^ = 0.09, F (6602) = 10.52, *p* < 0.001. After controlling for gender, family economic situation, and age, harsh parenting (β = 0.28, *p* < 0.001, LLCI = 0.20, ULCI = 0.36), peer support (β = 0.09, *p* < 0.05, LLCI = 0.01, ULCI = 0.17), and their interactions (β = 0.14, *p* < 0.001, LLCI = 0.06, ULCI = 0.21) were all significantly associated with negative coping styles. This indicates that peer support moderated the path between harsh parenting and negative coping styles.

Moreover, the model which tested harsh parenting, peer support, their interactions, and negative coping styles as predictors of life satisfaction was significant, R^2^ = 0.15, F (7601) = 15.76, *p* < 0.001. After controlling for gender, family economic situation, and age, harsh parenting was significantly negatively associated with life satisfaction (β = −0.10, *p* < 0.05, LLCI = −0.18, ULCI = −0.02). Peer support (β = 0.30, *p* < 0.001, LLCI = 0.22, ULCI = 0.37) and negative coping styles (β = −0.16, *p* < 0.001, LLCI = −0.24, ULCI = −0.09) were also significantly associated with life satisfaction. However, the interaction effect between harsh parenting and peer support was only marginally significantly associated with life satisfaction (β = 0.06, *p* = 0.10, LLCI = −0.01, ULCI = 0.14).

Harsh parenting was positively associated with negative coping styles, and negative coping styles were negatively associated with life satisfaction; thus, negative coping styles played a mediating role in the relationship between harsh parenting and life satisfaction. This finding supported H1.

**Fig. 2:**
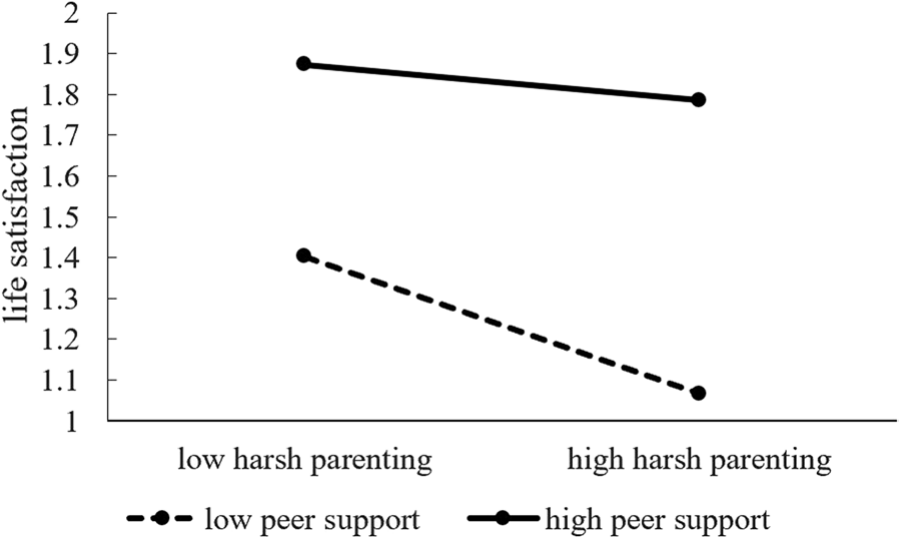
Moderation role of peer support in the relationship between harsh parenting and life satisfaction

To explain the moderating role of peer support in the association between harsh parenting and life satisfaction, we conducted a simple slope test. With peer support values of 1 SD below the mean and 1 SD above the mean, we calculated the effect of harsh parenting on life satisfaction. As shown in Fig. 2 a simple slope test indicated that for adolescents with high peer support, the association between harsh parenting and life satisfaction was not significant (β = −0.04, *p* = 0.48, LLCI= −0.15, ULCI = 0.07); whereas, for adolescents with low peer support, it was significant (β = −0.17, *p* < 0.01, LLCI = −0.27, ULCI = −0.06). Therefore, H2 was supported.

**Fig. 3:**
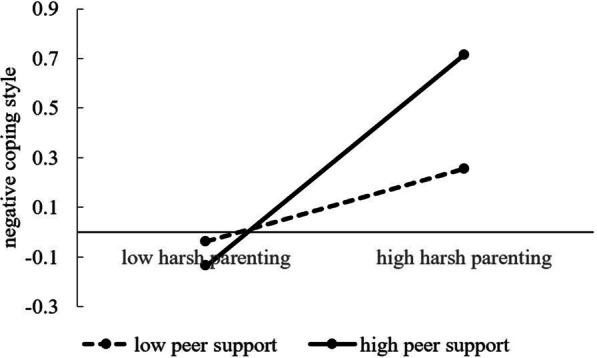
Moderation role of peer support in the relationship between harsh parenting and negative coping styles

Our results confirmed that peer support moderated the first path of the mediation effect (harsh parenting’s effect on negative coping styles). We conducted a simple slope analysis to explain the moderating effect of peer support on the association between harsh parenting and negative coping styles. We calculated the effect of harsh parenting on negative coping styles separately for low and high levels of peer support (1 SD below the mean and 1 SD above the mean). As shown in Fig. 3, the association between harsh parenting and negative coping styles was stronger for adolescents with high peer support (β = 0.42, *p* < 0.001, LLCI = 0.31, ULCI = 0.53) than for adolescents with low peer support (β = 0.14, *p* < 0.01, LLCI = 0.04, ULCI = 0.25). The moderating role of peer support in the relationship between harsh parenting and negative coping styles was verified, but peer support did not lessen the negative effect of harsh parenting. Thus, H3 was only partly supported.

We also analyzed whether the mediating role of negative coping styles in the relationship between harsh parenting and life satisfaction was moderated by peer support. The mediation effects were both significant when peer support was low and when it was high (β = −0.02, LLCI = −0.05, ULCI = −0.001; β = −0.07, LLCI = −0.12, ULCI = −0.03, respectively).

## Discussion

In this study, we found that harsh parenting in childhood was negatively associated with life satisfaction of Chinese college students, and this relationship can be explained by their negative coping styles. We also found that peer support mitigated the effects of harsh parenting on life satisfaction. These results provide a new perspective on the variability in responses of college students to environmental risk factors. The implications of this study could lead people to pay closer attention to the negative effects of harsh parenting, provide a better understanding of the mechanism of harsh parenting associated with Chinese late adolescents’ psychological development, and help to find targeted methods to improve the life satisfaction of late adolescents who are exposed to harsh parenting.

In addition, our results showed that male students experienced higher levels of harsh parenting, and less peer support and life satisfaction than female students. This might be because the gender stereotype emphasizes that men should be work-oriented, highly competent, independent, and focus on ability development and personal goals [37]. Males usually face greater social expectancies than females, especially in the Chinese culture. Therefore, they receive more harsh parenting. Meanwhile, according to gender stereotypes, women should be warm, gregarious, and focus on building social relationships [37]. This might result in females being more sociable and having better peer relationships and greater life satisfaction.

### The negative association between harsh parenting and life satisfaction

We confirmed the mediating role of negative coping styles in the relationship between harsh parenting and life satisfaction. Harsh parenting reflects parental dominance and absolute authority, as well as parental intrusiveness and over-control. This style of parenting is also characterized by a disregard for children’s psychological and physical needs [10]. Adolescents who have experienced harsh parenting might have negative parent-child interactions, cultivate negative self-perceptions, develop more negative explanations of and expectations for other people and situations, and have negative coping styles. Additionally, consistent with our hypothesis, we found that negative coping styles were negatively associated with life satisfaction. Individuals who adopt negative coping styles focus more on the negative aspects of events. They have fewer psychological resources and a lower capacity for finding sufficient and effective solutions to their problems [38]. They also live with the effects of negative events for a long time, are unable to effectively regulate emotions, and remain in negative emotional states; thus, indicating low levels of life satisfaction.

The results also showed that peer support moderated the direct relationship between harsh parenting and life satisfaction. Specifically, the negative association between harsh parenting and life satisfaction was significant only when college students had low levels of peer support. Thus, our results confirmed the interaction effect between different risk factors proposed by the bio-ecological model of human development [4]. Moreover, the results provided evidence of a stress-buffering effect, in which social support reduces the negative impact of harsh parenting. In general, the results of this study verify that different ecological factors have interacting effects on life satisfaction. A protective factor can weaken the negative effects of a risk factor.

The moderating role of peer support in the relationship between harsh parenting and negative coping styles was also documented, but the pattern of results was different from that of the moderating effect on the relationship between harsh parenting and life satisfaction. For college students who experienced a low level of harsh parenting, peer support appeared to be effective in reducing negative coping styles. However, for college students with high levels of harsh parenting, the provision of more peer support was unexpectedly associated with a more negative coping styles. We speculate that people who have experienced harsh parenting may be more likely to develop deviant peer relationships, and deviant peer support might correlate with more negative coping styles. Our research indicates that the role of peer support is complex, and peer support may not be a sufficient buffer in the context of high environmental risk.

### Limitations and future directions

Regarding the limitations and potential extensions of this study, several issues are worth noting. First, our measurements were all self-report scales, and although the test of common method bias was not determined to be of serious concern, it may be useful in future research to gather information about peer relations from peers. Moreover, reports of harsh parenting may show a social desirability bias. Furthermore, we measured students’ subjective family economic situations, investigated objective economic income would have higher credibility. In addition, many measurement subscales were used in this study. More comprehensive measurements of these factors are required.

Second, our data are correlational and do not allow for inferences about causality. Third, the representativeness of the sample population might be insufficient due to the lack of randomization and the small sample size. Fourth, in this study, we assessed peer support as a moderator of the effects of harsh parenting on negative coping styles and on life satisfaction based on the stress-buffering model. However, peer relationships may also mediate the association between parenting and developmental outcomes [27]. The negative association between harsh parenting and peer support may affect the moderating role of peer support.

Future research can expand our study in the following areas. Harsh parenting by fathers and mothers may affect adolescents’ psychological and behavioral development in different ways and with different intensities [10]. In this study, we did not distinguish between college students’ perceptions of their mothers’ and fathers’ harsh parenting. Moreover, physical and verbal aggression have different associations with adolescents’ developmental outcomes [39]. Further studies could divide harsh parenting into two dimensions to explore the differences in influence. In addition, adolescents’ attributions of harsh parenting could influence its’ negative effects. Misguided conclusions such as “My parents’ behavior is good for me” and “My parents just aren’t good at expressing their love” may serve to reduce the immediate negative psychological impact of harsh parenting. Finally, there are cultural differences in parenting practices and the influence of different parenting practices on adolescent development. Future research should focus on the role of harsh parenting outside the West, and it is necessary to systematically compare the impact of harsh parenting on Eastern and Western adolescents.

This study has several important practical implications. First, reducing harsh parenting is important for improving adolescents’ life satisfaction. Many family intervention programs have recognized the importance of parenting styles in promoting youth adjustment. However, harsh parenting is still a common practice in China because it is effective in inducing compliance in the short term. Chinese parents may have experienced harsh parenting and then carried these parenting practices to the next generation. Practical work should promote parents’ understanding of adolescents’ psychological needs and the negative effects of harsh parenting on adolescent development. Second, we found that negative coping styles were risk factors associated with lower life satisfaction. Training in emotion management, transformation of negative emotions, changing cognitive patterns, and acquiring adaptive coping styles may be important for improving life satisfaction. Furthermore, peer support could alleviate the negative effects of harsh parenting. Schools and society should play an important role in creating a social environment that is conducive to positive peer relations. Finally, ecological subsystems do not function independently but are interrelated. The improvement of adolescents’ life satisfaction relies on the positive interactions between different systems such as parents and peers. Interventions targeting multiple factors are more likely to achieve positive outcomes [40].

## Data Availability

The datasets generated during the current study are available at https://doi.org/10.6084/m9.figshare.20454468.v1.
